# Effect of Selenate on Viability and Selenomethionine Accumulation of *Chlorella sorokiniana* Grown in Batch Culture

**DOI:** 10.1155/2014/401265

**Published:** 2014-01-29

**Authors:** Živan Gojkovic, Carlos Vílchez, Rafael Torronteras, Javier Vigara, Veronica Gómez-Jacinto, Nora Janzer, José-Luis Gómez-Ariza, Ivana Márová, Ines Garbayo

**Affiliations:** ^1^Algal Biotechnology Group, Department of Chemistry and Material Sciences, Faculty of Experimental Sciences, University of Huelva, Campus el Carmen, Avenida de las Fuerzas Armadas s/n, 21007 Huelva, Spain; ^2^Department of Food Technology and Biotechnology, Faculty of Chemistry, Brno University of Technology, Purkyňova 118, 61200 Brno, Czech Republic; ^3^Algal Biotechnology Group, International Centre for Environmental Research (CIECEM), Parque Dunar s/n, Matalascaňas, Almonte, 21760 Huelva, Spain; ^4^Department of Environmental Biology and Public Health, University of Huelva, Campus el Carmen, 21007 Huelva, Spain; ^5^Department of Chemistry and Material Sciences, University of Huelva, Campus el Carmen, 21007 Huelva, Spain; ^6^University of Applied Science, Giessen, Wiesenstrasse 14, 35390 Giessen, Germany

## Abstract

The aim of this work was to study the effect of Se(+VI) on viability, cell morphology, and selenomethionine accumulation of the green alga *Chlorella sorokiniana* grown in batch cultures. Culture exposed to sublethal Se concentrations of 40 mg*·*L^−1^ (212 **μ**M) decreased growth rates for about 25% compared to control. A selenate EC_50_ value of 45 mg*·*L^−1^ (238.2 **μ**M) was determined. Results showed that chlorophyll and carotenoids contents were not affected by Se exposure, while oxygen evolution decreased by half. Ultrastructural studies revealed granular stroma, fingerprint-like appearance of thylakoids which did not compromise cell activity. Unlike control cultures, SDS PAGE electrophoresis of crude extracts from selenate-exposed cell cultures revealed appearance of a protein band identified as 53 kDa Rubisco large subunit of *Chlorella sorokiniana*, suggesting that selenate affects expression of the corresponding chloroplast gene as this subunit is encoded in the chloroplast DNA. Results revealed that the microalga was able to accumulate up to 140 mg*·*kg^−1^ of SeMet in 120 h of cultivation. This paper shows that *Chlorella sorokiniana* biomass can be enriched in the high value aminoacid SeMet in batch cultures, while keeping photochemical viability and carbon dioxide fixation activity intact, if exposed to suitable sublethal concentrations of Se.

## 1. Introduction

Selenium is a trace element that acts either as an essential micronutrient or as a toxic element for fish, birds, animals, and humans depending on its concentration [1, 2]. It is of fundamental importance to human health, it plays a role in mammalian development [3], immune function [4], and in slowing down aging [5]. At low levels, it contributes to normal cell growth and function (a daily intake of 40 *μ*g for adult men and 30 *μ*g for women is recommended by WHO [6]). High concentrations are toxic, causing the generation of reactive oxygen species (ROS), which can induce DNA oxidation, DNA double strand breaks, and cell death [7]. The primary cause of Se deficiency that reduces growth, reproduction, and even causes death is its low amount in soil and consequently in animal feed [8]. Selenium bioeffects are mainly involved in immune function, reproduction, metal toxicity resistance, and other biological functions [9]. Besides, selenium has been proven to be an effective anticancer agent mainly based on statistical and model studies [10], when it is supplied in a suitable bioactive form [11–13].

In nature, inorganic selenium is present in three oxidation states: selenate (+VI), selenite (+IV), and elemental selenium (0) over a range of natural water chemical conditions. Selenate is the dominant dissolved form, representing more than 67% of the total dissolved selenium concentration. Both selenates and selenites are taken up by microalgae and converted to protein-bound selenocysteine (SeCys) and selenomethionine (SeMet) [14]. SeCys is the predominant selenoaminoacid in tissues when inorganic selenium or organic bound as Se-enriched yeast is given to animals [15, 16]. Besides, selenium can substitute sulphur in methionine and forms SeMet. This can be incorporated unspecifically into proteins instead of methionine. SeMet cannot be synthesized by higher animals and humans [17] and is present in plant foods, while SeCys is more common in animal foods. Recent studies have shown that certain Se-compounds, as SeMet, are effective chemoprotective agents, reducing the incidence of breast, liver, prostate, and colorectal cancers in model systems [15, 18, 19]. Because SeMet is the main natural selenium form, synthesized SeMet or SeMet-enriched foods (e.g., selenized yeast) are acceptable as more effective forms of selenium in humans and animals [20, 21]. Some studies have been done to test the effects of Se-enriched animal feed in animal health, which has increased interest for plant enrichment in selenocompounds [19, 22, 23]. Microalgae appears to be the easiest plant-like biomass to be Se-enriched.

Many studies concerning Se toxicity in microalgae can be found in the literature; selenate effect has been studied in *Chlamydomonas reinhardtii* [1, 24–26], *Scenedesmus quadricauda* [27, 28], and cyanobacterium *Spirulina platensis* [29]. Similar studies have been done with *Chlorella zofingiensis* with emphasis on heat-stable selenoproteins [30], on metabolism of Se volatile compounds [14], Se effect on *Chlorella sp.* cultivated on glucose [16], and Se effect on continuous microalgae cultures of *Chlorella pyrenoidosa* [31] and *Chlorella sorokiniana* [32].

Microalgae *Chlorella sorokiniana* was selected for this study as an ideal target microorganism which is ubiquitous, exerts positive effects on human health and biotransforms selenate in selenocompounds such as SeMet [16, 33]. Study of selenate effect was focused on several levels: monitoring various culture parameters and comparing results with those of unexposed cultures (control cultures), ultrastructure examined by transmission electron microscopy, isolation and identification of Se-affected proteins, and Se biotransformation to SeMet and other Se aminoacids. To our knowledge, there are no recent detailed papers describing Se effect on microalgae batch cultures. With this study we offer new insight on SeMet-enriched *Chlorella sorokiniana* biomass production in batch cultures exposed to sublethal Se concentrations, intending to show that SeMet-enriched algal biomass production is feasible in batch systems, keeping both cell photochemical viability and structural stability if suitable selenium conditions are selected.

## 2. Materials and Methods 

### 2.1. Microalga, Growth Medium, and Experimental Conditions

The microalga *Chlorella sorokiniana* CCAP 211/8 K was obtained from the UTEX culture collection. It was maintained in modified M-8 medium [34] in Erlenmeyer flasks at 25°C and 165 *μ*mol photons m^−2^s^−1^. The culture medium was prepared as follows (composition expressed in g·L^−1^): KH_2_PO_4_, 0.74 g·L^−1^; Na_2_HPO_4_ × 2H_2_O, 0.26 g·L^−1^; MgSO_4_ × 7H_2_O, 0.4 g·L^−1^; CaCl_2_ × 2H_2_O, 0.013 g·L^−1^; KNO_3_, 3 g·L^−1^; EDTA ferric sodium salt, 0.116 g·L^−1^; Na_2_EDTA × 2H_2_O, 0.0372 g·L^−1^; H_3_BO_3_, 6.18 × 10^−5 ^g·L^−1^; MnCl_2_ × 4H_2_O, 1.3 × 10^−2 ^g·L^−1^; ZnSO_4_ × 7H_2_O, 3.20 × 10^−3 ^g·L^−1^; and CuSO_4_ × 5H_2_O, 3.2 × 10^−3 ^g·L^−1^. Chemicals were purchased from Sigma-Aldrich (Germany), unless otherwise indicated. In the prepared fresh medium precalculated amount of selenium was added in the form of aqueous stock solutions of Na-selenate (Na_2_SeO_4_). Prior to experiments, cultures were inoculated with cells in the exponential growth phase in order to obtain an initial cell density of approximately 3·10^6^ cell·mL^−1^. The pH was adjusted to 6.7 with concentrated solution of NaOH.

 The microalga *Chlorella sorokiniana* was cultivated in 5 L laboratory glass bottles at 25°C and continuously illuminated with white fluorescent lamps (Philips TLD, 30 W, 160 *μ*mol photons m^−2^s^−1^), at the surface of the flask. The irradiance was measured with a photoradiometer Delta OHM, model HD 9021, Italy. The culture suspension was mixed both with magnetic stirrer at 150 rpm and by air bubbling containing 5% (v/v) CO_2_, as unique carbon source. In order to cover a complete algal growth cycle, culture parameters were monitored three times a day. Selenate concentrations in the culture medium used in the experiments were 40 mg·L^−1^ selenate, for Se effect on culture growth and SeMet accumulation studies and 40 mg·L^−1^ and 100 mg·L^−1^ for cell ultrastructure studies.

### 2.2. Biomass Concentration and Optical Density

Biomass concentration was determined by dry weight measurements. Dry weight was determined by filtration of the culture broth over glass fiber filters with a pore size of 0.7 mm (Whatman GF/F, Kent, UK). The filter weight was determined on a 0.01 mg precision balance. Aliquots of 5 mL of culture broth, diluted 10 times with prefiltered demineralized water in order to remove inorganic salts, were filtered through prewashed, predried, and preweighed filters. Filters were dried at 80°C during at least 16 h and cooled down in a dessicator for 2 hours. Dry weight, expressed as g·L^−1^ of culture broth, was calculated by differential weight.

Optical density was determined spectrophotometrically at 680 nm using UV/Visible spectrophotometer (Ultrospec 3100 pro, Amersham Pharmacia Biotech, Uppsala, Sweden).

### 2.3. Population Density, Algal Growth, and Statistical Analysis

Population density was determined by counting the number of cells using a Neubauer chamber and light microscopy (Olympus CX41), and calculated based on the equation: *N* = 0.25 · 10^4^ · (∑*N*_*i*_) · *D* and expressed as 10^6^ cell·mL^−1^. Where *N* is population density (cell·mL^−1^), ∑*N*_*i*_ is total sum of the counted cell numbers on Neubauer chamber (*i* = 1,2, 3,4), and *D* is applied dilution of the culture.

To study the effect of Se on the algal growth, a logistic model, defined by Verhlust [35], was used. This model uses three key parameters: initial cell density at time zero (*N*_0_, cell·mL^−1^), maximal density that can theoretically be reached (*N*_max⁡_, cell·mL^−1^), and maximal culture growth rate (*μ*_*m*_, h^−1^). According to the model, cell density, *N*(*t*), at any time, (*t*), is given by the following equation:

(1) $$\begin{array}{c} N\left(t\right)=N_{\max}\cdot N_{0}\cdot {\left(N_{0}+\left(N_{\max}-N_{0}\right)\cdot e^{-\mu t}\right)}^{-1}. \end{array}$$

Maximal cell densities and growth rates were assessed by means of a logistic curve estimation function of SPSS Statistical Package software (v.19). Model curve was fitted to mean values of population density data [36], this model was previously used to describe growth kinetics of microalgae, in general [37] and of species such as *Chlamydomonas reinhardtii* [24–26] and *Chlorella minutissima* [38]. EC_50_ value is defined as half of the maximal effective concentration of a given substance; this is to say, concentration that provokes 50% of maximal effect. In this paper, EC_50_ for selenate is equivalent to 50% of *Chlorella sorokiniana* growth inhibiting selenate concentration. In order to calculate EC_50_ value, maximal growth rates obtained from growth curves previously fitted to logistic model were used to construct the dose-response curve. In order to predict EC_50_ we fitted growth rates data to the parameter log-logistic model (also known as Hill's model), thoroughly explained elsewhere [24–26] using the open source software “R statistical package” [39], according to instructions on fitting a single dose-response curve published by Ritz and Streibig [40]. R package has been previously used by Geoffroy et al. for statistical analysis of data on selenate effect on *Chlamydomonas reinhardtii* [1].

### 2.4. Chlorophyll and Carotenoids

The chlorophyll and carotenoids content was determined by methanol extraction and spectrophotometry. After centrifugation (5 min at 4400 rpm), biomass was mixed with methanol and the mixture was placed in an ultrasound bath for 5 min to disrupt the pellet. Subsequently, mixture was incubated at 60°C first and then cooled at 0°C to break the cells. After centrifugation, supernatant was collected and analyzed by UV/Visible spectrophotometry. Modified Arnon's equations [41] were used to calculate the chlorophyll and carotenoid concentrations in the extracts. The cell contents of chlorophyll and carotenoids were expressed per gram of biomass, calculated based on samples dry weight.

### 2.5. Measurement of Fluorescence

Another method used for assessing biological activity of the algal population was fluorometry. Maximum fluorescence yield (*Y*_op_) was determined by pulse amplitude modulation (PAM) fluorometry with the saturating-pulse technique. A chlorophyll fluorometer (PAM-210, Walz, Germany) was used. The samples were first adapted to dark for 15 min in order to open all photosystems reaction centers. Light of 0.04 *μ*mol photons m^−2^s^−1^ was used to measure the zero fluorescence level (*F*_0_). Saturating light pulse (1850 *μ*mol photons m^−2^s^−1^) was used to measure the maximum fluorescence (*F*_*m*_). Then the sample was illuminated with actinic light and series of saturating pulses in order to reach steady (light-adapted) state fluorescence (*F*′) and steady state maximum fluorescence *F*_*m*_′ level. Finally, the actinic light and saturating pulses were switched off to measure *F*_0_′ level. The maximum photochemical yield and effective photochemical yield of photosystem II were calculated using the equations [42]: *Y*_op_ = (*F*_*m*_ − *F*_0_)/*F*_*m*_ and Φ_PSII_ = (*F*_*m*_′ − *F*′)/*F*_*m*_′.

### 2.6. Oxygen Evolution

The photosynthetic activity was measured to test cell viability; 1 mL of algal cell culture was placed into a Clark-type electrode (Hansatech, UK) to measure O_2_ evolution. The electrode was equipped with a stirrer bar, a pressure corrector, and a temperature sensor. It was placed in a photosynthetic cylindrical chamber of 15 mm inside diameter and 10 mL capacity, surrounded by an outer water jacket for constant temperature operation. Measurements were made at 25°C under saturating white light (1500 *μ*mol photons m^−2^s^−1^) or darkness (endogenous respiration).

### 2.7. Cell Protein Isolation and Fractionation with Ammonium Sulfate

Cultures containing 40 mg·L^−1^ of selenite, as well as untreated culture, were grown in batch for 240 h. One liter of each culture was sampled on time zero and 120 h of cultivation as well as at the end of the experiment (240 h). Cells from sampled culture were collected by centrifugation (4400 rpm for 5 min) and resuspended in 20 mM phosphate buffer (pH 7) to a final concentration of 0.67 g·mL^−1^. Cell disruption was performed on ice with an ultrasonic probe (Lab Sonic) at 40% of power for 10 seconds, followed by a 50 second pause to avoid heat denaturation. This procedure was repeated 10 times. Extracts were centrifuged (13000 rpm for 20 min at 4°C), cell debris was discarded, and supernatant was collected. Prior to ammonium sulfate fractionation, nonprotein materials were precipitated with 0.1 M streptomycin sulfate solution in phosphate buffer (pH 7). Ammonium sulfate fractionation procedure was performed using protocol described by Harris [43]. All solid fractions were resuspended in 20 mM P-buffer (pH 7) and kept frozen until electrophoresis was performed.

### 2.8. SDS-Polyacrylamide Gel Electrophoresis (SDS-PAGE)

SDS-PAGE was performed by the method of Laemmli (1970) [44]. Protein samples were mixed with the sample buffer (0.5 M Tris-HCl, pH 6,8 containing 5% SDS, 20% glycerol) at 1 : 2 ratio at the presence of 10% *β*-mercaptoethanol. Electrophoresis was performed on 10% resolving gels with 4% stacking gels. Molecular weight marker 14.2–66 kDa (Sigma) was used to estimate the molecular weight of proteins. Volume of 20 *μ*L of sample was loaded in each well containing 15 *μ*g of proteins. Protein concentration was determined by spectrophotometry using BioRad Bradford reagent at 595 nm, with bovine serum albumin as the standard [45]. Electrophoresis run at 180 V for 75 min. Gels were washed three times with distilled water, stained with Coomassie-Blue stain for 180 min, and destained overnight with 10% acetic acid/30% ethanol aqueous solution.

### 2.9. Protein Analysis by MALDI-TOF-TOF Mass Spectrometry

Samples were automatically digested with trypsin according to standard protocols [46]. MALDI-TOF-TOF analysis was performed by Central Services of Research at the University of Cordoba, Spain, using an ABI Applied Biosystems 4700 Proteomics Analyzer (Amersham Biosciences). Mass spectra were obtained using a laser (337 nm, 200 Hz) as desorption ionization source. Data were acquired in the reflection positive mode using delayed extraction. Spectra were calibrated using trypsin autolysis products as internal standards. After MS acquisition, the 10 strongest peptides per spot were selected automatically for MS-MS analysis.

Identification of proteins was carried out by searching against NCBI nonredundant protein sequence database. MASCOT searching engine (Matrixscience, UK) was used for protein identification. Protein identifications with the score value higher than 60 were positively assigned, after considering MW and pI values.

### 2.10. Extraction and Determination of Selenium Species

Cultures of *Chlorella sorokiniana *were centrifuged to separate the pellet from the medium. Liquid nitrogen was applied to the pellet to disrupt the cell walls and an amount of 0.020 g was weighted in a centrifuge tube, then 0.02 g of Protease XIV was added. The extraction was performed with the assistance of a ultrasonic probe at 25% power during 2 minutes. After the extraction, the sample was centrifuged for 5 minutes at 6000 rpm and the supernatant collected. Finally the supernatant was filtered through 0.45 *μ*m (PVDF) filters and injected in the HPLC-ICP-MS.

The Se was measured by ICP-MS using the following operational conditions: forward power 1500 W, sampling depth 7-8 mm, auxiliary gas flow rate 0.10–0.15 mL·min^−1^, extract I: 0–3 V, extract II: −137,5 V, omega Bias-ce −20 V, omega Lens-ce −1.6 V, cell entrance −40 V, QP focus −15 V, cell exit −44 V, octP RF 190 V, octP bias −18 V, H_2_ flow 3.8 mLmin^−1^, QP bias −16 V, discriminator 8 m V, and analog HV 1840 V. The ^77^Se, ^80^Se, and ^82^Se were monitored for analysis, but only isotope ^80^Se was used for quantification. A solution containing Li, Y, Tl, and Ce (1 *μ*g·L^−1^ each) prepared in the mobile phase was used to tune the ICP-MS for sensitivity, resolution, percentage of oxides, and doubly charged ions. The chromatographic separation was performed on the basis of previously described instrumental coupling [47, 48].

### 2.11. Intracellular Structure Examination by Transmission Electron Microscopy (TEM)

For observations in electron microscopy, cultures containing 40 mg·L^−1^ and 100 mg·L^−1^ of selenate, as well as untreated culture, were cultivated in batch for 240 h. The algal cells were then collected from each culture, washed with culture medium, and collected by centrifugation (2500 rpm, 5 min). The algal cells were fixed with 1% glutaraldehyde in 0.1 M sodium cacodylate buffer (pH 7.4) for 2 h at 4°C. The cells were then washed three times (5 min each one) using the same buffer. The samples were postfixed with 1% osmium tetroxide in 0.2 M cacodylate buffer at 4°C for 1 h. Samples were washed with the same buffer, dehydrated in a graded ethanol series, and embedded in Epon 812 (EMbed 812 Kit; Electron Microscopy Science, Hatfield, PA, USA). Ultrathin sections of 80–90 nm obtained by an ultramicrotome (UCT, Leica, Wetzlar, Germany) and placed on nickel grids were stained with aqueous 1% (w/v) uranyl acetate and lead citrate. Transmission electron micrographs were observed with a JEM 1011 (JEOL Ltd., Tokyo, Japan) electron microscope using an accelerating voltage of 80 kV. Several photographs of entire cells and of local detailed structures were taken at random, analyzed, and compared to investigate selenium effect in the different subcellular structures of *Chlorella sorokiniana*. All chemicals used for histological preparation were purchased from Electron Microscopy Sciences.

### 2.12. Statistics

All experiments were triplicate unless indicated otherwise. Mean values of data are reported with standard deviations (±SD). Statistical analyses were performed using the Statistical Package for Social Sciences, SPSS v. 19 for Windows (SPSS Inc. USA) and open source software “R statistical package” propriety of R Development Core Team [39].

## 3. Results and Discussion

### 3.1. Effect of Selenate on Culture Growth

**Figure 1:**
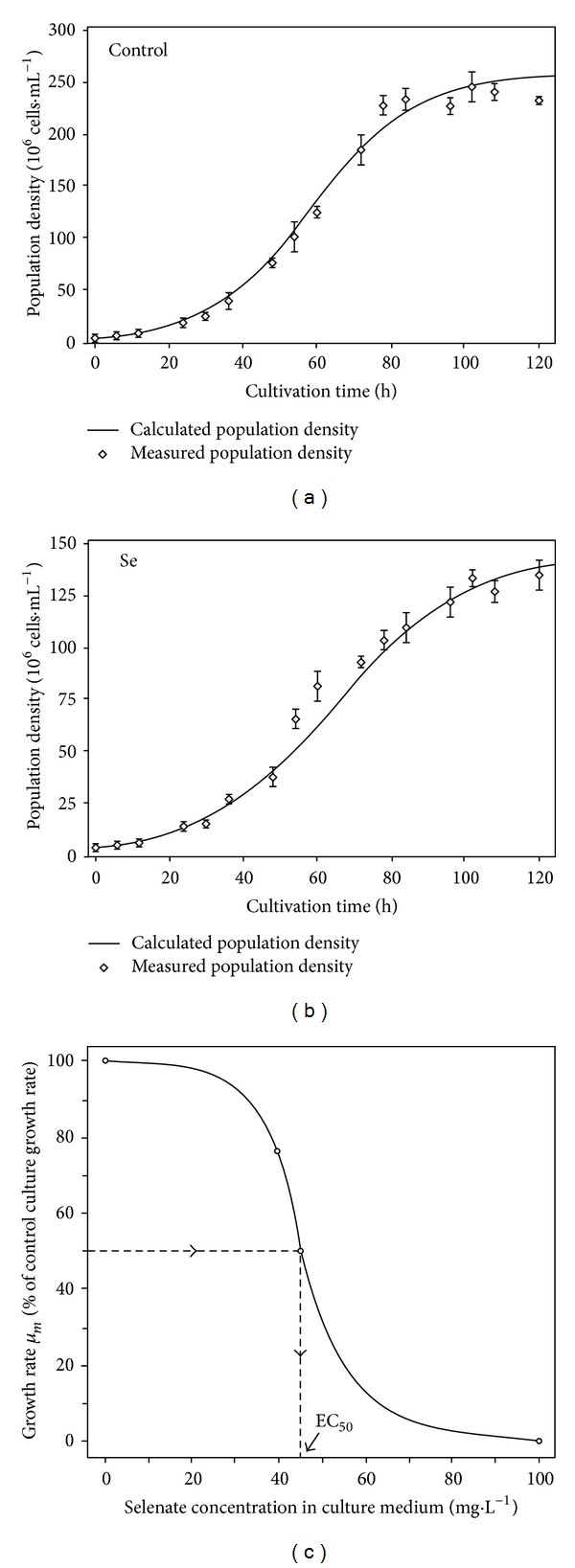
Growth curves of *Chlorella sorokiniana* culture with 40 mg·L^−1^ of selenate (a) and control culture (no selenate) (b). Data are given as mean values ± S.D. of the means. Both experimental data of population numbers and data calculated from logistic mathematical model fit are graphically presented in Figures 1(a) and 1(b). Model equation used is *N*(*t*) = *N*_max⁡_ · *N*_0_ · (*N*_0_+(*N*_max⁡_−*N*_0_)·*e* ^−*μt*^)^−1^, where *N*_0_ is the initial cell density at time zero, *N*_max⁡_ is the maximal density that cell population can theoretically reach in the indefinite time, *μ*_*m*_ is and the maximal culture growth rate (h^−1^), and *t* is cultivation time (day). Figure 1(c) presents concentration-response relationship between maximal growth rates from logistic growth models and selenate concentrations in medium. Concentration-response curve was fitted to the 2 parameter log-logistic model (Hill's model) using open source software “R statistical package.” To predict EC_50_ presumptions were made that concentration of 100 mg·L^−1^ selenate corresponds to the maximal (100%) growth inhibition, while zero inhibition corresponds to Se-free culture growth rate. Obtained EC_50_ value form the log-logistic model curve was 45 mg·L^−1^ of selenate.

The above described logistic mathematical model was used to fit data of population density changes as a function of time (Figure 1). Correlation coefficients (*R* ^2^) of the fitted models were 0.977 for selenite-exposed cultures and 0.974 for control cultures. Values of the parameters used in model are presented in Table 1. Experimental data of cell numbers and those data calculated from the model are graphically presented in Figures 1(a) and 1(b).

Maximal culture growth rate (*μ*_*m*_, day^−1^) is a fundamental growth parameter and if any key metabolic process of the cell is affected by toxins it will result in decreased *μ*_*m*_ values, which makes such parameter a relevant indicator for Se toxicity on microalgal cultures [31]. Maximal growth rate in 40 mg·L^−1^ selenate-exposed *Chlorella sorokiniana* culture (*μ*_max⁡_, 1.72 day^−1^, Table 1) accounted for 76% of the control value, which is comparable to the literature data. Continuously cultivated *Chlorella pyrenoidosa* cells showed *μ*_max⁡_ values of 1.46 and 0.94 day^−1^ for 0.53 and 1.41 mg·L^−1^ selenate, respectively, in culture medium, which represented 82% and 53% of the control growth rate value [31]. For the microalga *Selenastrum capricornutum* cultivated with 40 mg·L^−1^, Ibrahim and Spacie found a growth inhibition of 45% and a linear relationship between growth inhibition and selenate concentration [49]. In *Chlamydomonas reinhardtii* cells with 11.5 *μ*M of selenate in the culture medium, growth rate decreased only 5% compared to control cells [26]. Therefore, toxic effect of selenate greatly depends on microalgae genus and species. Consequently, prior to production of Se-enriched biomass, specific toxicity analysis for the selected algal species will be required.

Based on data published by Morlon et al. for *Chlamydomonas reinhardtii* cultivated in batch systems with 10 to 50 *μ*M selenite, EC_50_ values vary significantly from one experiment to another, which authors attributed to variations among different batch cultivations (see Table 2) [24, 25]. Toxic effect of Se can be expressed as EC_50_ value, a measure of the increase in biomass over time, and it is determined from the exponential phase [1, 26, 28, 31, 36, 50]. In our experiments with *Chlorella sorokiniana*, having in mind that 100 mg·L^−1^ selenate strongly inhibited cell growth and provoked severe cell deformation and death, as proven by ultrastructure microscopy, maximal (100%) growth inhibition was set for 100 mg·L^−1^, and the obtained EC_50_ value from the log-logistic model curve was 45 mg·L^−1^ (Figure 1(c)). In Table 2, EC_50_ values for different algae species are compared, expressed as Se concentrations (in both chemical forms, selenate Se(+VI), and selenite Se(+IV)) that provoked half of the maximal inhibitory effect, as published in the related literature (Table adopted from Vítová et al. [28]).

**Figure 2:**
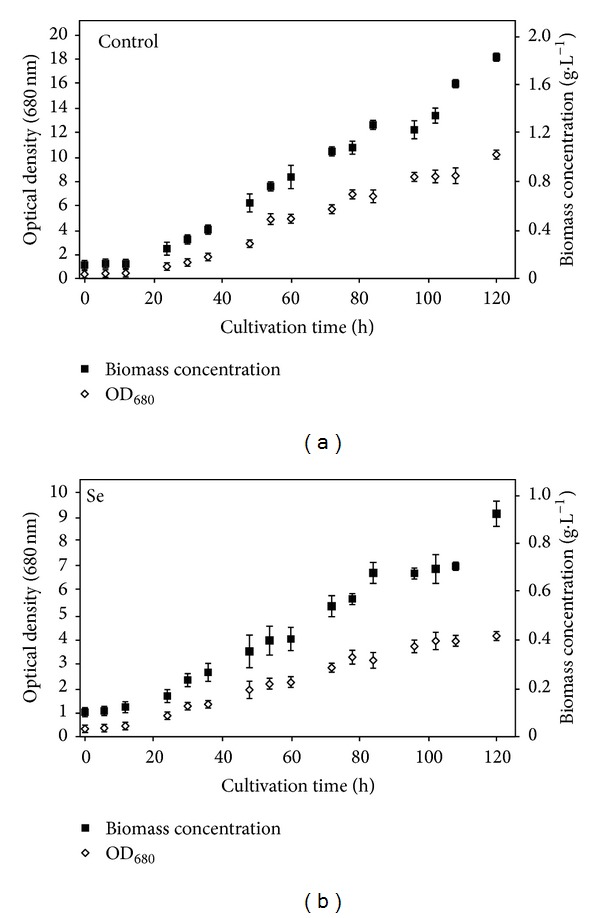
The optical density and biomass concentration of *Chlorella sorokiniana* as a function of cultivation time for (a) control (no selenate) and (b) culture with 40 mg·L^−1^ of selenate in culture medium. Data are given as mean values ± S.D. of the means.

In order to obtain SeMet enriched biomass while maintaining cell viability, a sublethal selenate concentration of 40 mg·L^−1^ was used. Time-course evolution of biomass concentration and optical density for control culture and Se-added cultures are presented in Figures 2(a) and 2(b). Data show that both optical density and biomass concentration decreased for about 50% compared to control culture values, and cultures were viable up to 120 h of cultivation.

In the literature, Se concentration range used in experiments varies significantly depending on microalgae species. Pelah and Cohen reported that *Chlorella zofingiensis* was resistant to selenite concentrations up to 100 mg·L^−1^ [30]. Li et al. found selenium to be an essential trace element at low concentrations and toxic at high levels in cyanobacterium *Spirulina platensis*, for which growth was enhanced when cultivated on 0.5 to 40 mg·L^−1^ selenate [29]. Umysová et al. observed that most of *Scenedesmus quadricauda* wild-type strain cells died within one or two days of cultivation if cultivated with Se (both selenate and selenite forms) at concentrations higher than 50 mg·L^−1^ [27]. Related studies on *Scenedesmus quadricauda* revealed that 50 mg·L^−1^ selenate in medium was not lethal to microalga cultures as cells grew and divided normally [28]. In our case, a selenate concentration of 40 mg·L^−1^ (212 *μ*M) in the culture medium was selected based on previously published data [32].

### 3.2. Effects of Selenate on Photosynthesis and Pigment Production

Chlorophyll fluorescence measurement is used as an economic and sensitive method for rapid detection of photoinhibition on algal cultures [42, 50–52].

**Figure 3:**
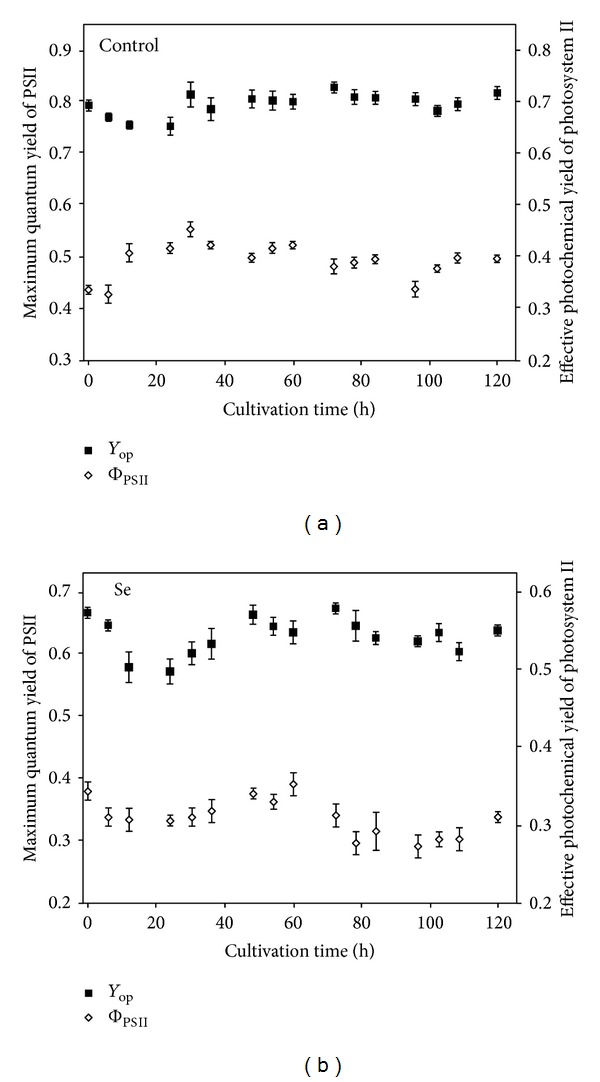
Maximum quantum yield of PSII (*Y*_op_) and effective photochemical yield of PSII (Φ_PSII_) in function of cultivation time for culture exposed to 40 mg·L^−1^ selenate in culture medium (a) and Se-free culture (b). Data are given as mean values ± S.D. of the means.

In Figures 3(a) and 3(b) it can be shown that for *Chlorella sorokiniana* cultures, 40 mg·L^−1^ (212 *μ*M) of selenate affected maximum quantum yield of PSII (*Y*_op_). Even though *Y*_op_ for Se-exposed cultures was approximately 20% lower than those control culture values, the obtained *Y*_op_ values were within the typical range for microalgae cells; therefore, cultures were accordingly considered viable throughout the experiment [53]. During the first 24 h of cultivation, values of *Y*_op_ for Se-exposed and control cultures decreased in approximately 15 and 10%, respectively, compared to initial values, due to culture adaptation phase. Nevertheless, this decrease was only temporary and *Y*_op_ values stabilized after 24 h, as previously found [32].

During the experiment effective photochemical yield (Φ_PSII_) values for control remained within the 0.33–0.42 range, while Φ_PSII_ of Se-exposed cultures remained within the 0.27–0.34 range (Figure 3(b)). Throughout the experiment, 25% decrease in Φ_PSII_ was found for Se-exposed cultures compared to control cultures.

The photosynthetic light reactions are located in the thylakoid membrane of the chloroplast, forming a closed system of stacked membranes surrounding the intrathylakoidal space, the lumen, thus separating it from outer chloroplast's area, and the stroma [53]. The thylakoid membrane contains both PSII and PSI systems with their respective reaction centers, which are connected by a series of electron carriers. PSII complexes are usually located in the stacked thylakoid region and the grana, while PSI complexes are located at the grana margins, facing the surrounding stroma [1].

It has been suggested that sublethal selenate concentrations in the culture medium can damage thylakoid membrane structures, thus affecting photosynthesis by both impairing PS II function resulting in decreased *Y*_op_ and by limiting electron transport between PSII and PSI, with a decrease in Φ_PSII_ which inhibits photosynthesis and decreases growth rate [1]. In that respect, Geoffroy et al. reported a decrease of 22% in *Y*_op_ after 24 h of cultivation of *Chlamydomonas reinhardtii* cells growing in 9.3 *μ*M of selenite. After 96 h the decrease arose 66% compared to control culture values. Effective photochemical yield (Φ_PSII_) decreased 52% after 24 h and 18% after 48 h exposure. These results evidence strong inhibition of the photosynthetic electron transport [1]. In our results, photosynthetic activity was less affected.

**Figure 4:**
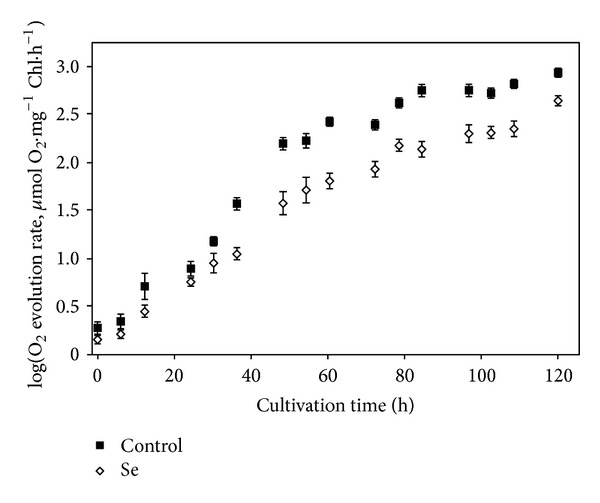
Photosynthetic oxygen evolution (*μ*mol O_2_ mg^−1^ Chl. h^−1^) by *Chlorella sorokiniana* in function of time for culture with 40 mg·L^−1^ of selenate and control culture (no selenate). Data are given as mean values ± S.D. of the means.

Oxygen evolution rates decreased 50% in cells exposed to 40 mg·L^−1^ Se compared to control cells after 48 h cultivation (Figure 4). That difference is similar to values reported by Schiavon et al. [54], in which a decrease of 44% compared to control cultures was determined for macroalga *Ulva sp.* after 10 days exposure to 100 mM selenite; once *Ulva sp.* thalli were transferred to fresh Se-free seawater, cultures restored to their original oxygen evolution rates [54].

**Figure 5:**
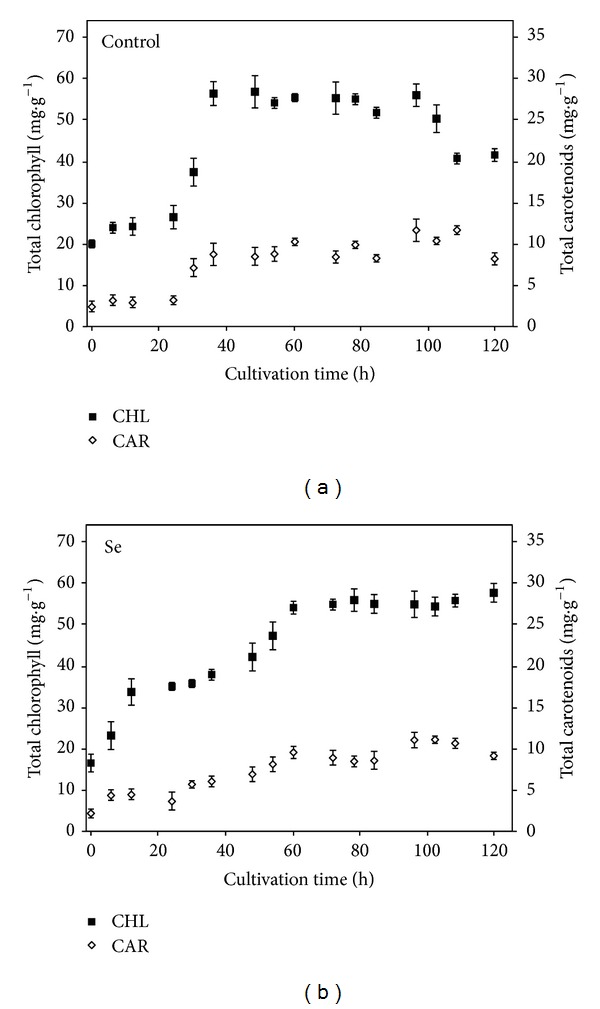
Total chlorophyll (mg·g^−1^) and total carotenoids (mg·g^−1^) content per biomass weight as a function of cultivation time for (a) control (no selenate) and (b) culture with 40 mg·L^−1^ of selenate in culture medium. Data are given as mean values ± S.D. of the means.

Total chlorophyll and carotenoid content of control culture (Figure 5) increased during the first 48 h of cultivation up to values of 60 mg·g^−1^ and 20 mg·g^−1^, respectively, and remained almost constant until 96 h of the experiment. From then on, total pigments content of control culture decreased due to self-shading effect (Figure 5(a)) [51, 55]. No significant differences in total pigment content were found for Se-exposed cultures. Based on PSII fluorescence, oxygen evolution, and pigment production data, it can be concluded that 40 mg·L^−1^ of selenate was a sublethal concentration for *Chlorella sorokiniana* culture and can therefore be used for SeMet accumulation studies. Similar findings for *Chlorella vulgaris* were reported in which chlorophyll production was not significantly affected by exposure to selenate during 9 days of batch cultivation [9].

### 3.3. Impact of Selenate on Ultrastructure of *Chlorella sorokiniana*

**Figure 6:**
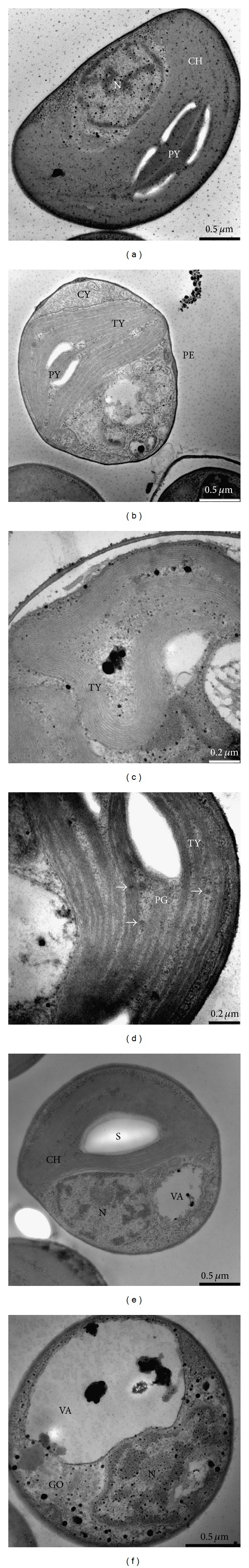
Ultrastructure images made by transmission electron microscopy of: Se-free single cell (a) and cell exposed to 40 mgL^−1^ selenate (b); fingerprint-like thylakoids (c) and plastoglobules (indicated by arrows) (d) in cell exposed to 100 mgL^−1^ selenate; autophagy in cell exposed to 40 mgL^−1^ selenate (e, f); CH: chloroplast; CY: cytoplasm; CW: cell wall; N: nucleus; PE: periplasm; PG: plastoglobules; PY: pyrenoid; TY: thylakoids; VA: autophagic vacuole; and V: vacuole.

Electron microscopy studies on the ultrastructure of *Chlorella sorokiniana* were carried out at the end of the experiment (240 h).  Figure 6(a) shows longitudinal and cross-sections through control cells (Se-free culture). This alga is about 3 *μ*m length and 2 *μ*m wide. The nucleus is about 1 *μ*m length and 1 *μ*m wide located in the central portion of the microalga. The cell has a prominent cup-shaped chloroplast that partially surrounds the nucleus, and the thylakoids inside are compressed and very dense which makes them indistinguishable. The pyrenoid (PY) is surrounded by four layers of starch. At 40 and 100 mg·L^−1^ of selenate in the culture medium, the stroma of the chloroplast became granule and less dense, and the thylakoids had a fingerprint-like appearance (Figures 6(b) and 6(c)). Fingerprint-like appearance of the chloroplast in Se-exposed microalga cultures was previously observed and reported in the related literature [1, 28]. Analysis of chloroplast ultrastructure by electron microscopy in cultures incubated with selenate 40 and 100 mg·L^−1^ revealed the presence of lipoprotein particles called “plastoglobules” in the stroma of chloroplasts that appeared as small black globules in close proximity to thylakoids (Figures 6(b), and 6(d)). Plastoglobules are involved in stress responses. Several studies have reported their presence in chloroplasts from plants grown under diverse stress conditions [56, 57]. Plastoglobules observed in cells treated with Se (Figure 6(d)) are plastid-localized lipoprotein particles that contain tocopherols and other lipid isoprenoid derived metabolites of commercial value, as well as structural proteins [58, 59]. In addition to vascular plants, plastoglobules are found in nonvascular species such as moss [60] and algae [61]. Some publications show that plastoglobules contain enzymes involved in the metabolism of secondary metabolites, as well as enzymes of unknown function [62]. At the highest Se concentration added to the culture medium (100 mg·L^−1^), some of the Se-exposed cells had large vacuoles (V) (Figure 6(e)) indicating a process of autophagy, a housekeeping mechanism, in which damaged or unwanted cellular components get degraded in vacuoles.

Autophagic vacuole (VA) and its compounds get recycled [63]. Structure of the cells exposed to 100 mg·L^−1^ became severely disrupted and normal cell organelles were often hardly distinguishable at the end of the experiment (Figure 6(f)). Therefore, from the results in can be inferred that 100 mg·L^−1^ selenate or higher concentrations are not compatible with cell viability, in good agreement with the biochemical results showed above in this paper.

### 3.4. Impact of Selenate on Total Protein of *Chlorella sorokiniana*

**Figure 7:**
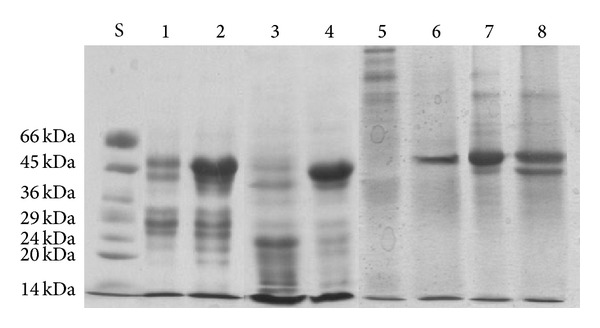
SDS-PAGE analysis of proteins extracted from biomass of *Chlorella sorokiniana*: 50% ammonium sulfate fractions of control (lanes 1 and 3) and 40 mg·L^−1^ selenate-exposed (lanes 2 and 4) cultures, 60% ammonium sulfate fractions of control (lane 5), 40 mg·L^−1^ selenate-exposed (lane 6) cultures, 40% ammonium sulfate fractions of control (lane 7), and 40 mg·L^−1^ selenate-exposed (lane 8) cultures. All lanes were extracted from cells of *Chlorella sorokiniana* after 120 h of cultivation. Lanes 1 to 4 origin from initial cultivation, while lanes 5 to 8 belong to repeated cultivation. Lanes 1 and 2 were loaded with 15 *μ*g of proteins, lane 3 with 20 *μ*g, lane 4 with 10 *μ*g, while lanes 5 to 8 contained 7.5 *μ*g of proteins each. S = protein ladder (molecular weight marker).

Electrophoresis of total protein isolate fractions revealed a band of approximately 50 kDa present in 50% ammonium sulfate fraction of Se-exposed cells, while the same band was absent in control culture fractions (Figure 7—lanes 1, 2). In order to locate in a more precise way that fraction where such a protein band appears in Se-exposed culture, the experiment was repeated and the results confirmed (Figure 7—lanes 3, 4). Total protein extract was fractionated with ammonium sulfate in the range of 30%–70%. After SDS PAGE electrophoresis, protein band of Se-exposed culture appeared in 60% ammonium sulfate fraction, while the same band was absent again in Se-free culture (Figure 7—lanes 5, 6). To identify proteins in Se-treated culture, bands were cut from SDS PAGE gels and analyzed by MS-TOF-TOF mass spectrometry (see Materials and Methods). Proteins from these bands were identified as 53 kDa large subunit of *Chlorella sorokiniana* Rubisco enzyme, suggesting that Se may interfere with proteins located in the chloroplast. Rubisco (ribulose 1,5-bisphosphate carboxylase/oxygenase), the most abundant enzyme in nature and responsible for CO_2_ fixation by photosynthetic organisms, is a complex protein composed from eight identical large subunits (*M*_*r*_ 53000) that are encoded in the chloroplast genome, each one with catalytic site and eight identical small subunits (*M*_*r*_ 14000) that are encoded in the nuclear genome [64]. Having in mind that selenate strongly affects chloroplast morphology and function [1, 26, 28], these results open the possibility that Se exposure could modify large subunit of *Chlorella sorokiniana* Rubisco by incorporation or association to it, as it is encoded in the chloroplast DNA. Se enters the microalga cell by competing with S metabolism and finally getting incorporated into aminoacids (SeMet and SeCys) [14]. Therefore, Se-aminoacids biosynthesis and its further incorporation into proteins might be among the biochemical reasons that explain the appearance of that probable Se-protein band.

Binding of Se on microalga proteins is reported by various authors [16, 30, 65, 66]. Boisson et al. described an increase of cytosolic selenium and total cell protein of the marine microalga *Cricosphaera elongate* when selenium concentrations in the culture increased, suggesting that these proteins take part in detoxifying process [65]. *Spirulina platensis* could accumulate 85% of selenium in organic form of which 25% was integrated with proteins [29]. Novoselov et al. identified selenoproteins present in a 20 to 80% ammonium sulfate fraction of *Chlamydomonas reinhardtii* with molecular weight of 7 to 52 kDa [66].

In spite of these results mentioned above, the mechanism of interaction between Se and large subunits of Rubisco is still unknown and merits further investigation.

### 3.5. Accumulation of SeMet during *Chlorella sorokiniana* Batch Cultivation in Presence of Selenate

**Figure 8:**
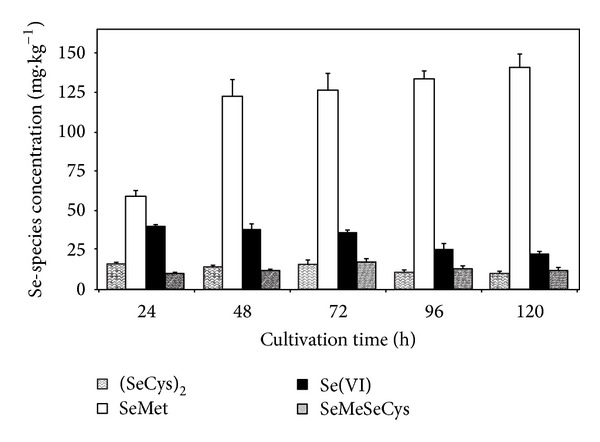
Selenium species concentration in *Chlorella sorokiniana* dry biomass (mg·kg^−1^) in function of cultivation time for culture exposed to 40 mg·L^−1^ selenate in culture medium. During cultivation 750 mL of culture volume was collected daily from each replicate bottle and centrifuged to obtain sufficient biomass to perform the necessary analyses. Data are given as mean values of at least three measurements ± S.D. of the means. Average difference between repeated measurements did not exceed 5% as can be seen from standard deviation indicators. The difference SeMet = selenomethionine; (SeCys)_2_ = selenocystine; SeMeSeCys = Se-methylselenocysteine; and Se(+VI) = selenate (SeO_4_^2−^).

As can be seen in Figure 8 *Chlorella sorokiniana* accumulated about 60 mg·kg^−1^ of SeMet in first 24 h of cultivation and doubled this value after 48 h, and increased moderately (approximately 5% daily), while intracellular Se(+VI) concentration gradually decreased, due to its transformation to SeMet and intermediates (SeCys)_2_ and SeMeSeCys [48]. At the end of the experiment total accumulation of SeMet was 140 mg·kg^−1^, while intracellular Se(+VI) concentration decreased by 50% from the initial value. Concentrations of intermediates SeMeSeCys and (SeCys)_2_ did not vary significantly throughout the experiments and maintained values between 10 and 20 mg·kg^−1^.

Neumann et al. found *Chlorella sp.* metabolized up to 87% of selenate to SeMet after 24 h of cultivation, and suggested that this microalga can develop an important capacity for rapid cellular conversion of selenate to SeMet in order to avoid toxic effect on long-term cell development [14]. More than 70% of the protein-bound Se in *Chlorella* biomass is found to be present in the form of SeMet, likely Se in Se-enriched yeast [16, 19]. Umysová et al. reported that wild type of *Scenedesmus quadricauda* accumulated 300 mg·kg^−1^ SeMet in the presence of 50 mg·L^−1^ of selenate [27]. Authors suggested that *Scenedesmus quadricauda* tolerance mechanism is an internal way to detoxify Se inside the cell [27]. Bottino et al. identified SeMeSeCys and SeCys amino acids present in *Chlorella* and *Dunaliella sp.* microalga exposed to 10 mg·L^−1^ selenite [67]. In our study *Chlorella sorokiniana* exposed to 40 mg·L^−1^ (212 *μ*M calculated as sodium selenate) accumulated 140 mg·kg^−1^ SeMet, which is approximately 20 mg·kg^−1^ more per 100 *μ*M of selenate than those values reported by Umysová et al. [27] for *Scenedesmus quadricauda* cells exposed to 50 mg·L^−1^ selenate (633 *μ*M calculated as elemental Se). Results obtained in this study proved SeMet to be prevailing selenoaminoacid accumulated by Se-exposed *Chlorella sorokiniana* culture.

## 4. Conclusions 

Microalga *Chlorella sorokiniana* was cultivated for 120 h in batch culture with sublethal selenate concentration of 40 mg·L^−1^ in order to evaluate the effect of selenate on culture growth, photosynthetic efficiency, cell ultrastructure, protein expression, and SeMet production. The goal was to prove that with this sublethal selenate concentration cultures were viable and able to accumulate significant amounts of SeMet in batch systems. Exposure of *Chlorella sorokiniana* to 40 mg·L^−1^ selenate decreased culture growth and oxygen evolution rates but had no effect on pigment content. Ultrastructural examination showed typical changes on chloroplast structure provoked by selenate exposure. Selenoproteins which appeared in the protein pool of Se-treated cells, but not in Se-free cells, were identified as 53 kDa large subunit of *Chlorella sorokiniana* Rubisco enzyme, suggesting that Se interferes with proteins located in chloroplast and might be incorporated into proteins as Se aminoacids. Microalga *Chlorella sorokiniana* exposed to 40 mg·L^−1^ selenate accumulated up to 140 mg·kg^−1^ of SeMet after 120 h of cultivation. Data obtained from this study open possibilities for larger culture volume trials in order to obtain biomass enriched in high value aminoacid SeMet.

## Figures and Tables

**Table 1:** Values of growth parameters *N*_max⁡_ (10^6^ cell·mL^−1^) and *μ*_max⁡_ (day^−1^) obtained by fitting logistic model equation to experimental data of population density in function of time from selenium exposed culture and control. Model curve was fitted to mean values of population density data (see Figure 1); hence, fitted models give single parameters values, instead of *N*_max⁡_ and *μ*_*m*_ mean values ± S.D.

Selenate concentration (mg·L^−1^)	0	40
*N*_max⁡_ (10^6^ cell·mL^−1^)	252	145
*μ*_max⁡_ (day^−1^)	1.72	1.31
Correlation coefficients (*R* ^2^)	0.977	0.974

**Table 2:** Values of EC_50_ parameter for microalga cultures exposed to Se salts, expressed as Se(+IV) and/or Se(+VI) concentrations (mg·L^−1^/**μ**mol·L^−1^) in culture medium, as determined and published in the literature. In some studies Se concentrations (in mg·L^−1^) were expressed based on weight of elemental Se, while in present study it was expressed based on weight of sodium selenate (Na_2_SeO_4_). Table adapted from Vítová et al. (2011) [28].

Reference	Microalga specie	EC_50_				Cultivation type Cont./Batch
		Selenate		Selenite		
		(mg·L^−1^)	(*μ*mol·L^−1^)	(mg·L^−1^)	(*μ*mol·L^−1^)	
Vítová et al. (2011) [28]	*Scenedesmus quadricauda *	33	418	4	50	B
Geoffroy et al. (2007) [1]	*Chlamydomonas reinhardtii *	0.36	4.5	—	—	B
Morlon et al. (2005a) [24]	*Chlamydomonas reinhardtii *	—	—	1.1	14	B
Morlon et al. (2005b) [25]	*Chlamydomonas reinhardtii *	—	—	6.3	80	B
Fournier et al. (2010) [26]	*Chlamydomonas reinhardtii *	0.032	0.4	—	—	B
Fournier et al. (2010) [26]	*Chlamydomonas reinhardtii *	0.245	3.1	—	—	B
Bennett (1988) [31]	*Chlorella pyrenoidosa *	0.79	10	—	—	C
Present study	*Chlorella sorokiniana *	45	238.2	—	—	B

## Nomenclature

*μ*: Specific growth rate (h^−1^)
*μ*_*m*_: Maximal specific growth rate (h^−1^)
Φ_PSII_: Effective photochemical yield of PSII
Car_tot_: Total carotenoids per dry weight of biomass (mg·g^−1^)
Chl_tot_: Total chlorophyll per dry weight of biomass (mg·g^−1^)
EC_50_: Selenate concentration at which growth rate has 50% of the unexposed culture value (mg·L^−1^)
OD_680_: Optical density measured at wavelength of 680 nm
PS II: Photosystem II electron carrier complex of thylakoid membrane
Rubisco: Ribulose 1,5-bisphosphate carboxylase/oxygenase enzyme
SeMet: Selenomethionine
(SeCys)_2_: Selenocystine
SeMeSeCys: Se-methylselenocysteine
Se(+VI): Selenate (SeO_4_^2−^);
*Y*_op_: Maximum quantum yield of PSII.
